# Web 3D for Public, Environmental and Occupational Health: Early Examples from Second Life^®^

**DOI:** 10.3390/ijerph5040290

**Published:** 2008-12-18

**Authors:** Maged N. Kamel Boulos, Rameshsharma Ramloll, Ray Jones, Susan Toth-Cohen

**Affiliations:** 1 Faculty of Health and Social Work, University of Plymouth, Drake Circus, Plymouth, Devon, PL4 8AA, UK; E-Mails: mnkamelboulos@plymouth.ac.uk (M. N. K. B.); ray.jones@plymouth.ac.uk (R. J.); 2 Idaho State University, Pocatello, USA; E-Mail: r.ramloll@gmail.com (R. R.); 3 Department of Occupational Therapy, Jefferson College of Health Professions, Thomas Jefferson University, USA; E-Mail: Susan.Toth-Cohen@jefferson.edu (S. T. C.)

**Keywords:** Public health, emergency preparedness, environmental health, occupational therapy, 3D virtual worlds, internet

## Abstract

Over the past three years (2006–2008), the medical/health and public health communities have shown a growing interest in using online 3D virtual worlds like Second Life^®^ (http://secondlife.com/) for health education, community outreach, training and simulations purposes. 3D virtual worlds are seen as the precursors of ‘Web 3D’, the next major iteration of the Internet that will follow in the coming years. This paper provides a tour of several flagship Web 3D experiences in Second Life^®^, including Play2Train Islands (emergency preparedness training), the US Centers for Disease Control and Prevention—CDC Island (public health), Karuna Island (AIDS support and information), Tox Town at Virtual NLM Island (US National Library of Medicine - environmental health), and Jefferson’s Occupational Therapy Center. We also discuss the potential and future of Web 3D. These are still early days of 3D virtual worlds, and there are still many more untapped potentials and affordances of 3D virtual worlds that are yet to be explored, as the technology matures further and improves over the coming months and years.

## 1. Introduction

Sivan [1] of the Metaverse1 Project Consortium (http://www.metaverse1.org/) defines three-dimensional (3D) virtual worlds as a ‘*3D3C*’ combination of:

– *3D*: controllable graphical environment (with 3D spatial audio) – one can zoom, change camera position, *etc.*;
– Community: real people can create groups, helped by a system that allows communities to blossom;
– Creation (or ‘plasticity’): there are ways to build and edit content, services, and various stuff; and
– *C*ommerce: the ability to attach real economic value (real money) to services, as necessary (*cf.* e-commerce and fund raising activities on the flat, conventional Web).

Over the past three years (2006–2008) the medical/health and public health communities around the world have shown a growing interest in using 3D virtual worlds like Second Life^®^ (http://secondlife.com/) for health education, community outreach, training and simulations purposes [2–18] (see also http://healthcybermap.org/sl.htm). 3D virtual worlds like Second Life^®^ and Twinity (http://twinity.com/) can be approached as 3D social networks, where people can also collaboratively create and edit various objects in the virtual world in real time (think of it as a ‘3D wiki’), besides meeting each other and interacting with existing objects [14]. These worlds are often seen to as the precursors of Web 3D, the next major iteration of the Internet (also sometimes called the Metaverse) that will follow in the coming years (2015–2020) [15, 16, 19].

The popular ‘Second Life in Education’ wiki (http://sleducation.wikispaces.com/educationaluses), and references [14, 17, 20] list dozens of uses of 3D virtual worlds, including but not limited to:

– Distance and flexible education;
– Presentations, panels and discussions;
– Training and skills development, including self-paced tutorials;
– Treasure hunts and quests;
– Role-plays and simulations;
– Data visualizations and modelling;
– Displays and exhibits, including immersive ones;
– Libraries, art galleries and museums;
– Multimedia and games design;
– Machinima (video clips produced entirely in the virtual world);
– Virtual tourism, cultural immersion and cultural exchange;
– Language teaching and practice, and language immersion;
– Awareness/consciousness raising and even fund raising by charities;
– Support and opportunities for people with disabilities (stroke, autism, *etc.*);
– Business, commerce, financial practice and modelling;
– Real estate practice (e.g., visit accurate property replicas in the virtual world);
– Product design, prototyping, user-testing and market research; and
– Urban/city and healthcare facilities planning and prototyping.

In the rest of this paper we will visit several flagship Web 3D experiences in Second Life^®^, including Play2Train Islands (emergency preparedness training), the US Centers for Disease Control and Prevention—CDC Island (public health), Karuna Island (AIDS support and information), Tox Town at Virtual NLM Island (US National Library of Medicine - environmental health), and Jefferson’s Occupational Therapy Center. We will then discuss the potentials and future of Web 3D.

## 2. Public, Environmental and Occupational Health Examples from Second Life®

### 2.1. Play2Train: a Virtual Learning Environment for Emergency Preparedness Training

Very few hospitals can afford to orchestrate the chaos and downtime that would result from a pandemic illness or other disasters through full scale live exercises. Traditionally, planning for such events is mostly limited to face to face discussions with staff, facilitated by emergency preparedness experts. Live exercises are too costly and most hospitals cannot stage them as often as needed to meet the training requirements for emergency preparedness. During live full scale exercises, the quality of care takes a hit because hospital staff is distracted and operates with strained resources under altered work flow conditions. A virtual training environment, however, provides realistic scenarios designed for specific learning objectives, can be accessed at point of need, has minimal footprint on real world operations, and does not put patients or any subjects directly at risk. Virtual learning environments for training have rarely been designed from the get-go to replace live exercises. Rather, their main role is to increase opportunities for diagnosing live exercise plans, for collaborative learning and above all for allowing geographically dispersed stakeholders to participate in a common emergency preparedness effort.

The Idaho Bioterrorism Awareness and Preparedness Program (http://www.ibapp.org/), which is funded by the Office of the Assistant Secretary for Preparedness and Response (http://www.hhs.gov/aspr/), created Play2Train (http://www.play2train.org/), a virtual hospital and town environment that supports exercises for emergency preparedness training. A major objective of IBAPP is to provide training at the point of need, and Play2Train illustrates one of the distance learning methodologies advocated by the programme.

#### 2.1.1. Play2Train design and implementation

The uniqueness of the Play2Train effort lies in the way the virtual environment was developed. It is the result of unanticipated and opportunistic collaborative interactions between geographically dispersed stakeholders within the very platform, the Second Life^®^ Grid, that is hosting it. The stakeholders include software designers, subject matter experts, such as risk communication experts, incident command instructors, health care providers and law enforcement personnel with relevant emergency preparedness expertise, and potentially anyone who is required to receive emergency preparedness training, including university or nursing students and health care providers. With limited resources, IBAPP was able to create a virtual training environment using the know-how of a loosely coupled community of subject matter experts and designers.

#### 2.1.2. The importance of plasticity

Participants access the Play2Train environment through the Internet. It is built on the Second Life^®^ Grid platform, a virtual world in which players assume the form of ‘avatars’—animated characters—to socialize, engage in activities and even buy and sell goods. Created by Linden Lab (USA), Second Life^®^ provides powerful user content-generation tools, meaning that the virtual environment can be shaped *in situ* by any individual or groups who log into it. For example, users can create buildings, vehicles or landscapes. Users’ ability to shape the virtual world they operate in through avatars is a property referred to as *plasticity*.

By packing social networking, content creation and content use within the same medium, Second Life^®^ provides opportunities for individuals to be both content users and creators—they can create or interact with content according to immediate needs. In our case, this means that subject matter experts have opportunities to dabble in content creation, build prototypes and scrutinize production processes led by a content design team or groups of design teams. These experts also can “drop in” any time to check on the training environment’s development progress and are in a better position to make actionable recommendations. In traditional environments, expert recommendations often cannot be implemented because of the experts’ lack of knowledge regarding the environment’s limitations. Thus, plasticity plays an important role in making expert participation in virtual environment development more effective.

The plasticity of the Second Life^®^ platform has provided Play2Train with three other undisputable gains. First, most of the active Play2Train collaborators emerged from the Second Life^®^ social network. Play2Train’s current status is proof of the effectiveness of a production process by and wholly contained within the Second Life^®^ platform.

Second, the training and tabletop scenarios are worked on during meetings held in the training environment. The recent introduction of spatial voice support, which enables avatars to communicate by speaking to each other, provides tremendous assistance in these activities. With spatial voice support, avatar speech sounds are rendered to mimic the behaviour of real-life sound sources. Avatars sitting around a table and speaking would experience the same voice soundscape as if they were sitting around a real-life table. In short, speech sounds from the speaker on the left will originate from the left, speech sounds from the speaker from the right will originate from the right and the volume of the voice indicates how far the speech source is [14].

Finally, the tabletop exercises developed in Play2Train can be modified easily, which enables instructors or content creators to improvise scenarios as needed. For example, an incident command system instructor can change the evolution of an exercise on the fly by introducing unanticipated secondary events that will require a rapid re-evaluation and re-adaptation of the incident command system in place. This flexibility is not found in most traditional, online, virtual-reality-based simulations; most have pre-defined scenarios. Those benefits are not only relevant to emergency preparedness training environments but to other educational domains as well.

Another element of Second Life^®^’s plasticity is users’ ability to create applications that mesh or “mash-up” with existing Web applications [15, 16]. Data exchanges between the Second Life^®^ platform and the rest of the Internet allow the exploration of blended, or mixed reality, learning paradigms. The Play2Train environment, for example, shows emergency preparedness videos in virtual classrooms to supplement classroom activities before tabletop exercises are carried out. Students have access to basic tools that allow them to document and push information about their activities in the training environment out to Web sites for viewing by non-users. Instructors have the opportunity to load quizzes that students can have access to on their own or in groups. A very active section of the Second Life^®^ education community has already made some progress on integrating a learning management system into the platform (http://www.sloodle.org/moodle/), and Play2Train is looking forward to adding this functionality to our training environment.

Debriefing, where the instructor has the opportunity to clarify students’ experiences and how they related to the tabletop exercise’s learning objectives, plays an essential role in simulation-based training. The Second Life^®^ platform allows the instructor to capture training activities as recorded animations or videos, also known as machinimas, and then play them back into the virtual environment for the instructor to watch with the students. All these activities are user-level operations and do not require assistance from Play2Train design or technical team members.

#### 2.1.3. Variety of scenarios, users

The Play2Train virtual infrastructure can support tabletop exercises dealing with topics such as incident command systems, risk communication, simple triage rapid transportation and the Strategic National Stockpile. This virtual infrastructure includes a hospital, a town, a uniform supply store offering uniforms for various relevant professions, a range of rapidly deployable medical facilities such as surge tents, mobile isolation units and emergency preparedness vehicles, and a set of applications to simulate fires, explosions and weather conditions. Unlike many learning spaces on the Second Life^®^ platform, the Play2Train environment is not publicly accessible.

One of the main challenges is to make the Play2Train environment accessible to a wide spectrum of users with varying comfort levels with computers and online games. Second Life^®^ already has already a large number of users; at any given time, 65,000 people will be logged in to access various virtual spaces (2008 figures). Many of our collaborators were the result of chance encounters with avatars who were already exploring this environment and also had similar ideas regarding possible applications that included emergency preparedness. These pioneers typically were highly functional within the Second Life^®^ environment, and they were able to participate more easily in our emergency preparedness training efforts.

Individuals new to the Second Life^®^ platform have to participate in our Play2Train orientation program, where they are explained the basics of navigation, communication and interaction in the virtual environment. After they complete the orientation, they are allowed to participate in the tabletop exercises. A shorter version of the orientation program is offered to some users who may already be familiar with the kind of interactions in virtual environments similar to those developed on the Second Life® platform.

#### 2.1.4. Examples of training activities in Play2Train

*Seton Hall University Mass Casualty Incident Exercise to support a Crisis Emergency and Risk Communication (CERC) course:* One of the primary challenges in teaching the CERC content and the accompanying management skills is to provide health management students with opportunities to simulate real life experiences. Meeting the JCAHO (Joint Commission on the Accreditation of Healthcare Organizations) objectives of creating an emergency preparedness plan and coordinating all those involved, such as major health institutions, community responders and public health departments, normally requires participation in an extensive tabletop exercise or conducting an institutional drill. Although, tabletop dioramas and accompanying exercises can be configured to portray the impact of a pandemic over time, additional activities would be needed to simulate community partner participation. Currently, very few opportunities exist to duplicate the “real life” practice of a TOPOFF (Top Officials) type exercise which provides a mock regional experience and includes multiple community stakeholders. To help the Master of Health Administration students at Seton Hall University learn the emergency and risk communication skills presented by CERC in a real-time scenario, an innovative learning option was selected. The students were provided with the opportunity to participate in a Play2Train scenario that was enhanced to integrate the emergency and risk communication elements.

**Figure 1:**
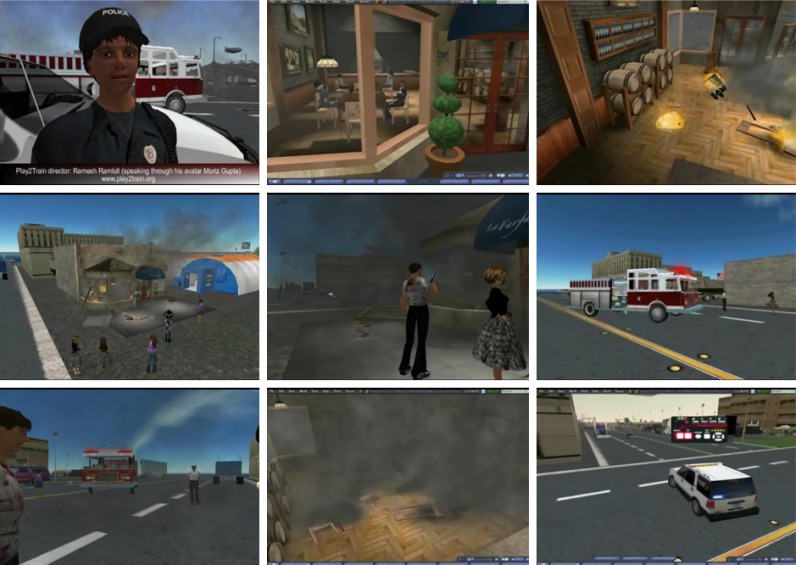
A mass casualty incident simulation to support the learning goals of a CERC course.

In the Play2Train virtual scenario (Figure 1), an emergency has occurred outside a town restaurant that has sent multiple persons to a local community hospital. At a moment’s notice and without background information, hospital management must respond both clinically and publicly to address the current situation. The scenario involves several different scenes including the hospital, an incident command centre, a triage station and a press communication room. Each of the participants are assigned a role and given a set of tasks to complete. Virtual roles include the following positions: Hospital Incident Commander, Chief Medical Officer, Emergency Department Director, Hospital Security Officer, Chief Communication Officer, Nurse Administrator/Supervisor, Facilities Manager, Communication Coordinator, Personnel Director and Registration Coordinator/Medical Records. Faculty and support personnel play the roles of the Public Health Director, EMS (Emergency Medical Services), Police and Fire personnel, and the local media. In their respective roles, students respond to the health emergency and work as a team to pool their information and complete the required communication messages following the principles outlined in the CERC training.

**Figure 2:**
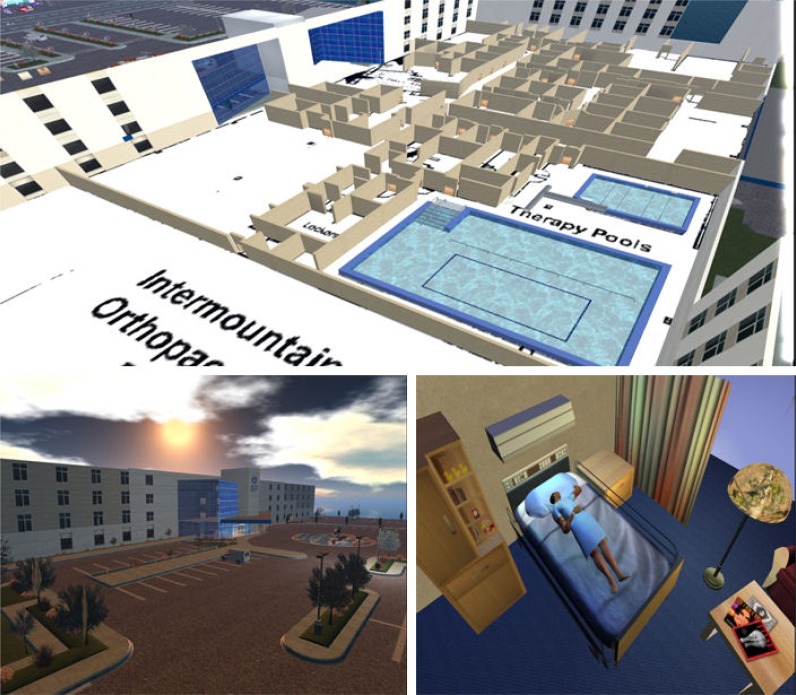
A realistic virtual replica of a real world hospital.

*Elks Rehabilitation Evacuation Exercise*: A virtual replica of the Elks Rehabilitation Hospital in Boise, Idaho, was developed to illustrate possible healthcare related applications to hospital staff. The replica was constructed based on floor plans that were made available to the designers of the virtual environment. When introducing a new collaborative learning medium such as this virtual learning environment, it is usually easier to present ‘use cases’ that make sense to the target audience especially when the virtual environment presented looks familiar (Figure 2). The virtual Elks Rehabilitation Hospital was used by participants to discuss, design, diagnose and evaluate evacuation plans during a fire emergency scenario which would impact access to various exit points.

**Figure 3:**
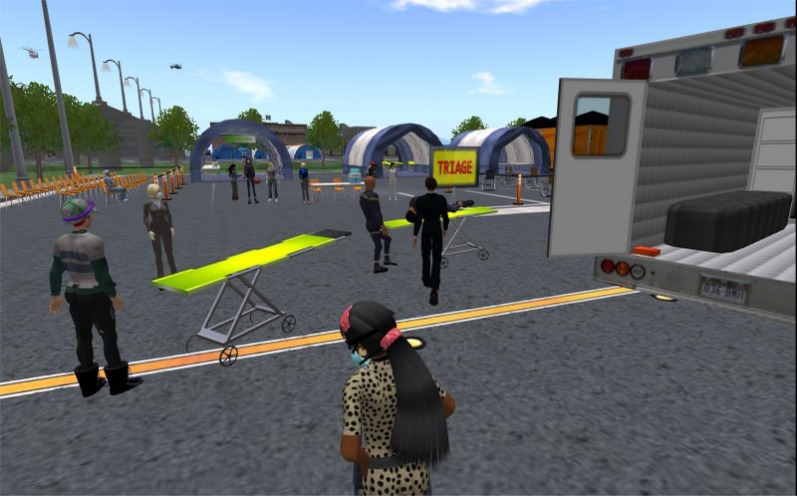
Assessing and triaging patients on arrival at the alternate health care facility deployed on a parking lot of a hospital.

*Bingham Memorial Hospital Pandemic Influenza Triage Exercise:* The objective of the Bingham Memorial Hospital Pandemic Influenza Triage Exercise was to provide an opportunity for hospital staff and participants from other relevant institutions such as the Blackfoot Police and Fire departments to take part in a virtual exercise in order to practise a response plan to address a pandemic influenza situation (Figure 3).

#### 2.1.5. Real-life payoff

There has been much interest in 3D, massive multiplayer environments for training because the virtual world takes up no physical space and can be accessed at a distance. Virtual training environments keep trainees or care receivers out of harm’s way, and they also provide better opportunities for fine-grained tracking of trainee activities.

Immersion—a gaming term that refers to a player’s engrossment in and focus on an environment (with ‘suspension of disbelief’ because of the realism of the environment and the interactions it provides)—is an important factor to consider when evaluating the effectiveness of virtual training environments. Well-designed virtual training environments have been shown to score high on immersion.

Until now, high implementation costs of virtual training environments have kept many training programmes at bay in spite of evidence of their growing effectiveness. The user content-generation opportunities in Second Life^®^ is attracting educators all over the world because of an increasing awareness that they can participate in the creation of the content and tools they need to whatever extent they want in this new medium. Play2Train is just one early example of an application developed on this platform.

As the popularity of this training methodology grows, we can expect to see many hospitals provide access to a virtual replica of itself though a menu item on their Web sites (e.g., this early example at http://virtualpalomarwest.org/). These virtual hospitals will provide a wide range of interaction possibilities—from patient education to telehealth applications—that go beyond being simple stages for emergency exercises.

### 2.2. US CDC Island: Engaging with Consumers ‘wherever they are’

In the 1970s health promoters in the ‘real world’ moved from providing information about the risks to health from lifestyle choices, to engaging with communities to try to bring about change. The equivalent paradigm shift is happening online. Until relatively recently, health services have approached online health education and support by providing good quality evidence based information trying to make this as accessible as possible. Issues of concern have included methods to assess quality of Web sites [21] and ways to ensure equity of access. Now service providers recognise that they need to engage with consumers ‘wherever they are’, either in terms of their thinking, or their characteristics, or in terms of their online location.

The need to present online information to patients or clients in ways which are tailored to their individual needs or characteristics as well as to their online preferences has long been recognised and there is an increasing body of evidence [22, 23] that tailored information can be more effective. Even a single individual can have multiple and variable online preferences that change at different times/settings, and also according to the Internet access devices/bandwidth available to them at various places [24]. Health providers have therefore sought ways of tailoring information and repurposing/repackaging it in multiple ways or “flavours” to reach out to the widest possible audiences. But health services have also recognised that in today’s social Web environment online activity is increasingly concerned with person-person support, peer communication and “viral” mass marketing in health promotion [25] than in accessing static information sources in solo, and so experiments in how to engage with different communities online are underway. Questions to be answered include finding the best approaches for patients, students and health professionals to learn with and from each other in a virtual world, and identifying how professionals can best learn about their virtual world clients and their needs, and contribute advice and support in a way that is not perceived as threatening or destroying the democratic basis of these online communities, all while ensuring the quality of these services.

In the same way that traditional mass media are fragmenting into smaller and more specialised sectors, channels and publications, online users have very varied practices and locations that continuously change and increase in number. For example, in November 2008 there were >57,000 Yahoo! Groups on ‘Health & Wellness’ (http://dir.groups.yahoo.com/dir/). But online gatherings are no longer limited to the flat Web. As online users move into greater use of 3D environments and virtual worlds, so those concerned with health promotion and health education need to explore those worlds too.

This diversity of ‘location’, either in the real or the online world, makes developing a communication strategy for health providers quite challenging, as they need to be present “everywhere at the same time”. On the one hand large groups of people have yet to go online. Use of all technologies decreases with age; for example, in 2007 in the UK 90 per cent of those aged 25–34 owned a digital TV compared to 58 per cent of those over 75, 99 per cent of those aged 16–19 owned a mobile phone compared to 42 per cent of those over 75, and 82 per cent of 16–19 year olds had access to the Internet compared to 10 per cent of those aged over 75 [26]. Amongst those who go online there is great variation in both the capacity and power of their device(s) and connection(s), as well as their ability to use different software and hardware [27], so that some can ‘manage’ simple email or perhaps Facebook (http://www.facebook.com/) while others are leading almost “virtual” lives in Second Life® or other 3D worlds.

In their ‘State of CDC FY 2006 Report’, the US Centers for Disease Control and Prevention (CDC) have a short section entitled ‘“Second Life” Lets CDC Be Everywhere—All at Once’, in which they briefly explain the main rationale for their presence in Second Life®, which has since then developed to a full 63,296 sq. m. island today in the virtual world (CDC Island: http://slurl.com/secondlife/CDC%20Island/191/87/22):

“Why Second Life? CDC’s work to advance public health means going where people are. As every virtual person represents a real person, CDC’s presence in Second Life offers yet another opportunity to learn and teach about public health. So, if there are thousands of people spending significant amounts of time online, where better to influence their health-related decision making? CDC messages of all kinds can be placed in this space for people to view passively or with which to become more actively engaged in efforts to improve their own health; and all of this in an environment *of their own choosing*.” [28]

**Figure 4:**
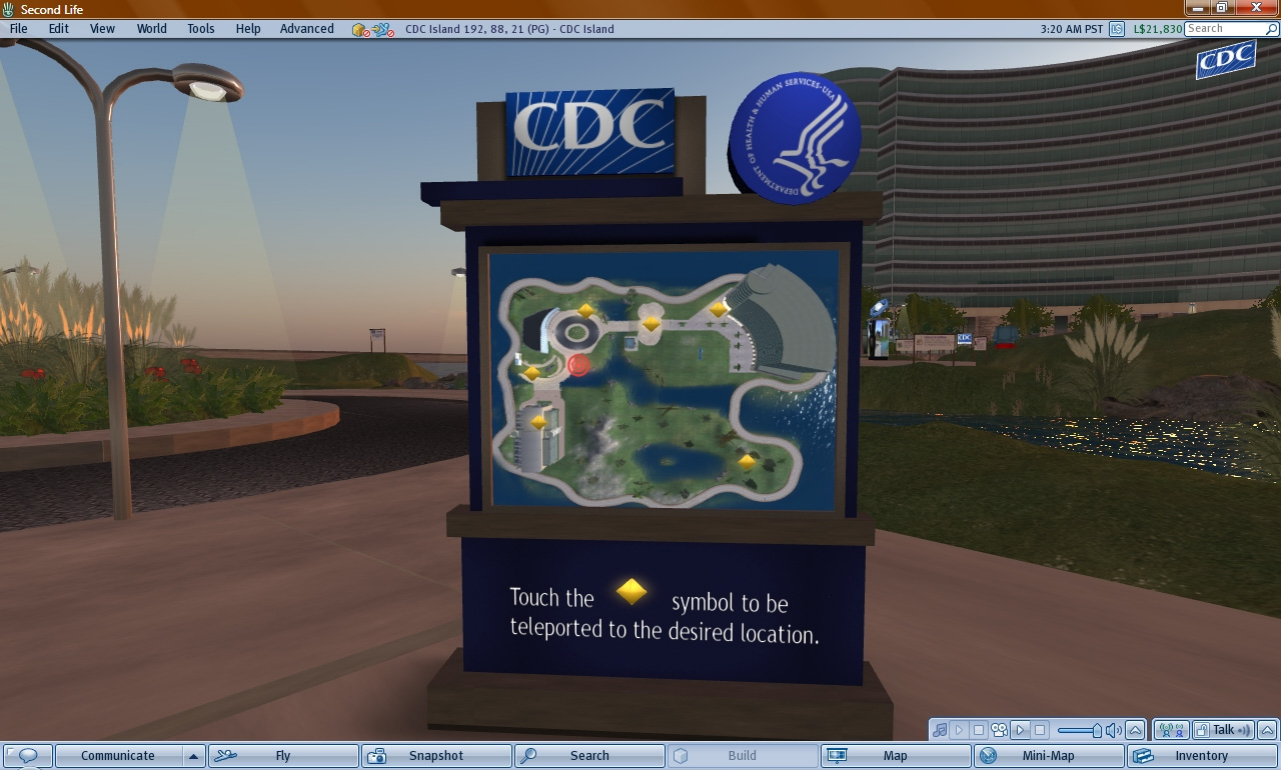
A teleport hub at the CDC Island in Second Life^®^ offering instant access (teleport) to the various places and buildings on the island.

Rather than wait for people to come to them (which might not always happen across different user groups), the CDC took the initiative to find out where those people are and meet them in those spaces. The US CDC presence in Second Life^®^ has been ongoing since July 2006 on a very small parcel of virtual land. In 2008, the CDC opened to the public a full island in Second Life^®^, the CDC Island (Figure 4), and vacated their earlier parcel. Unlike the latter, the new CDC Island much better matches online consumers’ expectations of a world’s leading public health agency of the calibre of the US CDC (cf. in flat Web terms, a single Web page vs. a full Web portal with its own domain name).

Before launching the CDC Island, the Division of eHealth Marketing at CDC conducted a research study to determine the direction of their new virtual island in Second Life^®^. Their goal was to expand their existing public health communication channels and to become an even greater trusted source of credible online health information. Helped by Engauge (http://www.engauge.com/), the scoping research involved Second Life^®^ avatars and their human operators who had characteristics that matched the CDC target Second Life audience. In total, the researchers conducted 25 avatar interviews and one five-avatar focus group. The study focused on finding out user needs, preferences, and expectations of a CDC Second Life^®^ Island, and identifying content, features, and functionality that would enable the virtual world presence to make the greatest real-world health impact [29, 30].

Results from this research were then used to build much of the new content and functionalities on today’s (2008) CDC Island. Among the many interesting findings of this study is the revelation that participants did not want to see things on an island (e.g., mere flat Web links) that they could easily see on a conventional Web site [29]. This is a very clear call for exploring and capitalising on the unique affordances of 3D virtual worlds as an online medium.

The CDC Island is professionally built with a number of interesting attractions, including an ‘issues of the month’ walk, a conference centre, a virtual health career museum, and a virtual lab. The island is nicely landscaped with refreshing picnic and open air meeting areas. Visitors might even come across one of the “walking kiosks” or robot kiosks that patrol the island. (These latter objects are attention-grabbing, moving information signs/dispensers “with legs”.) Also available is a private space where avatars can discuss with expert CDC staff (when available in-world) sensitive topics such as HIV/AIDS. Visitors will find podcasts on a range of health topics and can even examine actual slide images from the CDC’s public health library by looking through a virtual microscope in the virtual lab.

Second Life® is an excellent immersive environment for modelling health behaviours. Each of the interactive tools at the CDC Island is designed to influence healthy behaviours. The CDC also values the ability of peers to connect with one another, sometimes anonymously/privately as mentioned above, which also helps influence health and safety decisions by having the communities talking among themselves, out of which the CDC can come to conclusions that influence its decision-making.

Second Life^®^ is not the CDC’s first venture into virtual worlds nor its first attempt to engage visitors of these worlds in virtual behaviours that might influence real-world health decisions. In 2005, it partnered with Whyville (http://www.whyville.net/), a virtual community targeting children and early teens aged 8 to 15 to promote flu vaccine awareness [31]. They released a potent virtual flu virus that covered unprotected avatars with spots. During its first year in Whyville, the CDC virtually vaccinated 20,000 Whyville residents. During its 2007–2008 campaign, 41,000 residents were vaccinated. Because grandparents use Whyville to connect with their grandchildren, the CDC was also able to involve this important cohort in the awareness campaign (cf. “viral” marketing [25]). People who queue for the virtual vaccination become more motivated and thus more likely to actually go out and get actual vaccinations when indicated.

### 2.3. Karuna: AIDS Information and Outreach in the Virtual World of Second Life®

In Pali, an early Indo-Aryan language of India, Karuā embodies the desire to remove harm and suffering, and is paired with mettā, the desire to bring about the well-being and happiness of others. Funded by a US National Library of Medicine (NLM) grant, the US Alliance Library System (ALS—http://www.alliancelibrarysystem.com/article.cfm?id=383) is collaborating with AIDS.gov, NIDA (US National Institute on Drug Abuse—http://www.nida.nih.gov/) and other health agencies in the virtual world to develop Karuna Island (http://slurl.com/secondlife/Karuna/60/106/26 and http://www.karunasl.info/), a community library and resource centre for people of all ages diagnosed with AIDS, those that are HIV positive, their friends and families, caregivers, and those who provide them with health information such as librarians and health professionals.

**Figure 5:**
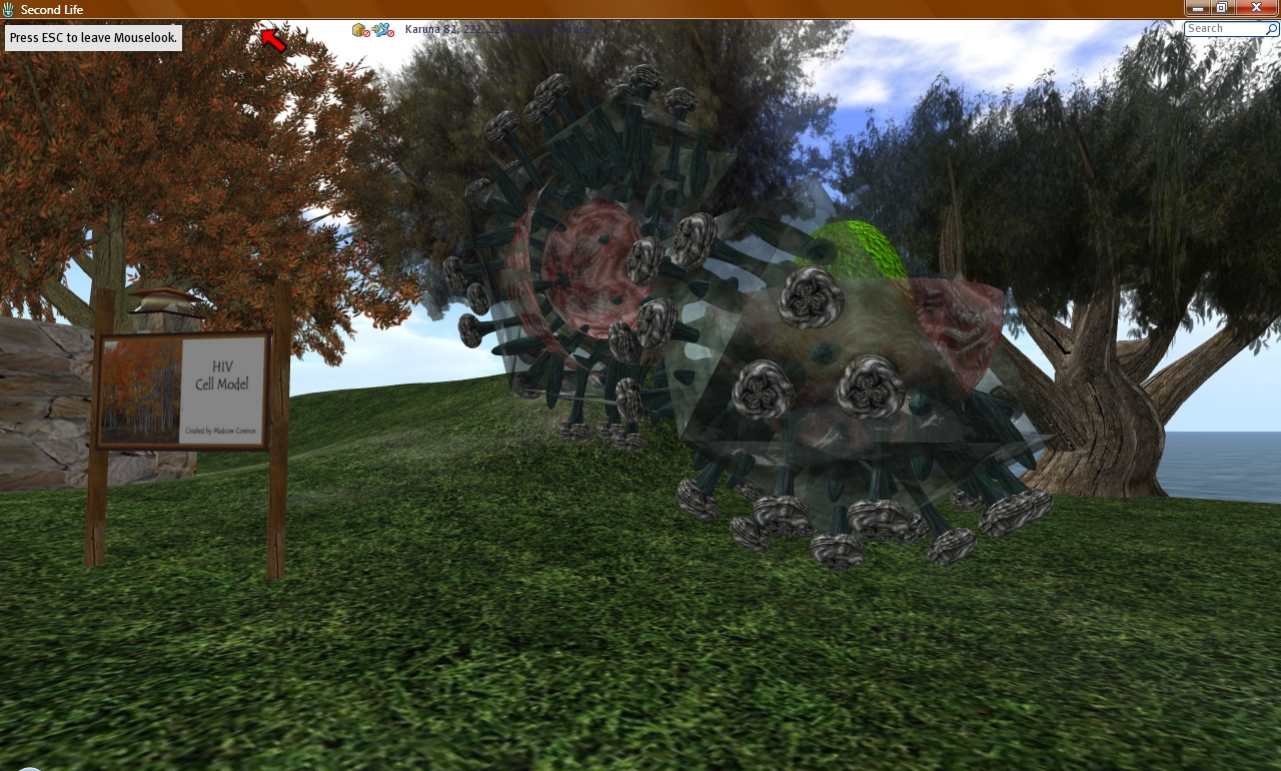
A 3D HIV Cell Model at Karuna Island.

Karuna was officially launched in Second Life^®^ on World AIDS Day 2008 (1^st^ December 2008— Figure 5). Its resource centre will improve awareness of the disease and promote quality health information about its different aspects. The space to provide these services in Second Life^®^ also includes a community centre, private spaces for smaller group meetings, an open-air auditorium for events, as well as a dedicated event centre with technology enhanced seminar-style learning. The library and resource centre are planned to contain a wealth of materials including tutorials, newsfeeds, links to current awareness searches in PubMed, links to other NLM resources about AIDS, AIDS.gov, and other relevant resources. Karuna’s ‘garden of experience’ is a place where narratives about the AIDS/HIV experiences of loss, adjustment, joy, and community spirit can be shared through art in the form of music, poetry, video, and more (see Karuna Island snapshots at http://www.flickr.com/photos/verdeo/3015625338/in/set-72157608590656238/).

Project objectives from the original NLM-funded Karuna Island proposal include:

– Building an AIDS information and community centre in Second Life^®^;
– Developing and compiling quality resources in the virtual library on AIDS/HIV;
– Providing informational displays on AIDS/HIV;
– Training AIDS/HIV patients, their families, and others on how to search for quality information on all aspects of the disease;
– Increasing awareness of NLM resources on AIDS/HIV; and
– Collaborating with other AIDS/HIV and health information agencies to provide quality health information in the virtual world.

**Figure 6:**
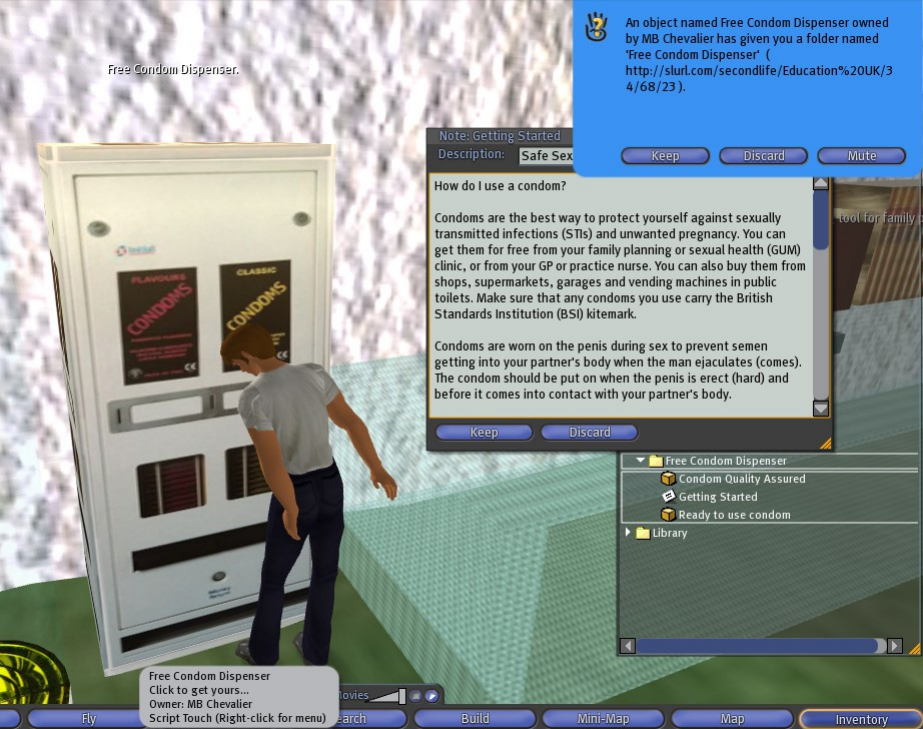
A ‘raising awareness’ edutainment artefact at the University of Plymouth Sexual Health SIM in Second Life^®^[13]. This free virtual condom dispenser dispenses two 3D male condom objects to user’s inventory, plus an information Notecard on how to use a (real) male condom.

It is noteworthy that Karuna Island project is building on, and linking to, a long history of sexual health public education and outreach activities in the virtual world of Second Life®, of which a leading example is the University of Plymouth Sexual Health SIM (simulator) project (http://healthcybermap.org/slsexualhealth/) described in [13] (Figure 6). (The University of Plymouth Sexual Health SIM now also has an experimental presence on the New World Grid, outside the Second Life^®^ Grid (see http://www.newworldgrid.com/ and http://healthcybermap.org/slsexualhealth/Eleniel.jpg).)

### 2.4. Tox Town at Virtual NLM: Environmental Health Information from the US National Library of Medicine

Located in the SciLands of Second Life^®^ (http://www.scilands.org/), Virtual Tox Town (http://slurl.com/secondlife/Virtual%20NLM/134/152/25) is a project of the Specialized Information Services (SIS) Division of the National Library of Medicine (NLM), National Institutes of Health, US Department of Health and Humans Services. SIS (http://sis.nlm.nih.gov/) is responsible for information resources and services in toxicology, environmental health, chemistry, HIV/AIDS, and specialized topics in minority health.

The flat Web-based version of Tox Town (http://toxtown.nlm.nih.gov/) allows for limited interactivity with chemicals and locations. It is an online interactive guide to commonly encountered toxic substances, health, and the environment, where users can explore a Port, Town, City, Farm, or US-Mexico Border community to identify common environmental hazards. Created in 2005, it is continually updated and is offered in both English and Spanish, with a special text version for assistive reading devices and software. Resources for teachers such as classroom activities and promotional items are also included. Tox Town subscribes to the HONcode principles (http://www.hon.ch/HONcode/Conduct.html?HONConduct779612).

NLM has re-created Tox Town in the virtual world of Second Life^®^ in an effort to expand the original concept of Tox Town from a two-dimensional (2D) conventional Web environment to a much richer, true 3D virtual town experience. By using a virtual world such as Second Life®, NLM is able to greatly enhance the interactivity of the application and bring it closer to a real life experience. Virtual worlds also offer the opportunity for several avatars to interact with each other and the environment.

Virtual Tox Town is designed to give visitors information on:

– Everyday locations where they might find toxic chemicals;
– Non-technical descriptions of chemicals;
– How the environment can impact human health;
– A range of related interactive objects and activities; and
– Links to selected, authoritative chemical information and resources on environmental health topics on the Internet.

Tox Town has two main themes: (1) toxic chemicals and locations where they may be found; and (2) environmental health concerns. NLM designed Virtual Tox Town to be similar to the Web version so users can learn about the chemicals in a specific location, or can choose a specific chemical and learn the locations in which the chemical may be found. Users can also learn about environmental health concerns at each location.

Chemicals included in Virtual Tox Town meet the following criteria:

– Toxic (or perceived toxicity of interest to general public);
– Commonly encountered in the United States;
– Known or expected to impact human health

Of interest to federal agencies that regulate, research, or advise on a chemical’s health effects. The US Environmental Protection Agency and the US Agency for Toxic Substances and Disease Registry are examples of Federal agencies concerned about toxic chemicals.

Chemicals or substances that are voluntarily ingested, such as drugs, dietary supplements, or caffeine, are not included.

**Figure 7:**
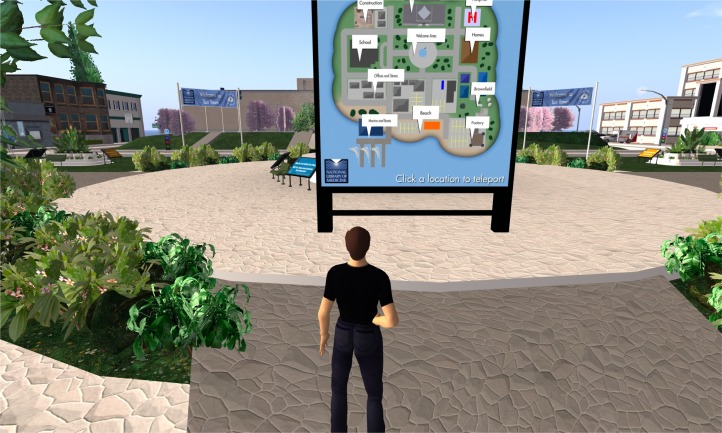
Tox Town’s HUD in Second Life®. The HUD can be repositioned around the user’s screen according to user’s preference.

Visitors can begin their exploration of Tox Town by taking the tour helicopter throughout the island for an introduction to the capabilities of Tox Town. The best way to experience Tox Town is to use the island’s Heads Up Display or HUD (Figure 7 – see also ‘What is a HUD?’ at http://secondlife.com/app/help/avatar/huds.php). The HUD will display all of the chemicals that are in each location and give users information about each chemical. It can also be used to play an interactive game where the user identifies chemicals using clues at incidents involving toxic chemicals. The HUD provides a handy link to the NLM’s WISER tool on the Web at http://wiser.nlm.nih.gov/ to help users find the solutions.

**Figure 8:**
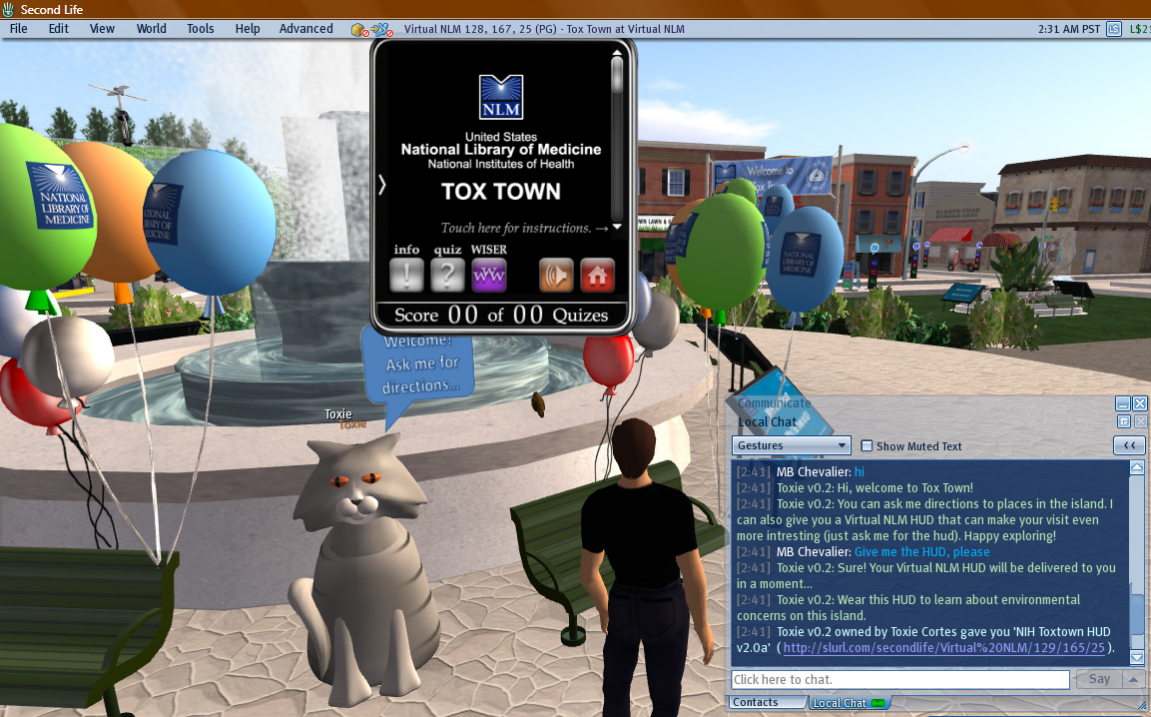
The teleport map in Tox Town’s Welcome Area.

The island provides several options to explore locations in Tox Town, the toxic chemicals they contain and the environmental health concerns they represent:

Users may look for the teleport map in the Welcome Area (Figure 8) and choose a location on the map to be teleported there, e.g., School, Beach, Factory, Brownfield, *etc.* There is also a teleport map for the ‘interactive chemical display’ which will show visitors all of the places a specific chemical might be found in Tox Town.

Users may also travel around Tox Town on their own. Each building that has information on environmental health concerns is labelled with a sign at street level and on its roof. Besides buildings, users may look for other objects, such as a dog, that also have information.

Like its flat Web version, the information in Virtual Tox Town on chemical and environmental concerns comes from the TOXNET (http://toxnet.nlm.nih.gov/) and MedlinePlus (http://medlineplus.gov/) resources of the US NLM, as well as other authoritative sources. Selection guidelines (based on MedlinePlus) are used in evaluating in-world links to Web pages. The chemical descriptions (for example, the in-world Notecard on ‘What is arsenic?’) were written for Tox Town based on TOXNET and other resources and were reviewed by NLM toxicology staff.

### 2.5. Jefferson’s Occupational Therapy Center: Educating the Public about Environmental Adaptations and Work Environment Risks

Because it is immersive and three-dimensional, Second Life^®^ and other virtual worlds afford unique opportunities to create environments that put forth new possibilities in everyday (non-virtual) life [32]. Such virtual worlds foster a sense of actual presence, of “being there” and may enable users to more clearly visualize options available to them in real life. One example of the ways that virtual worlds enable users to consider possibilities are virtual homes that demonstrate how persons with physical and mental challenges can live safer and more satisfying lives by adapting their home environments. Such homes have been constructed at the Occupational Therapy Center at Jefferson in Second Life^®^ (Jefferson College of Health Professions, Philadelphia, USA – overview video: http://www.youtube.com/watch?v=t1gFem4YnWA) and the eXtension project at Morrill Virtual State Fair that is associated with the US Cooperative Extension System.

**Figure 9:**
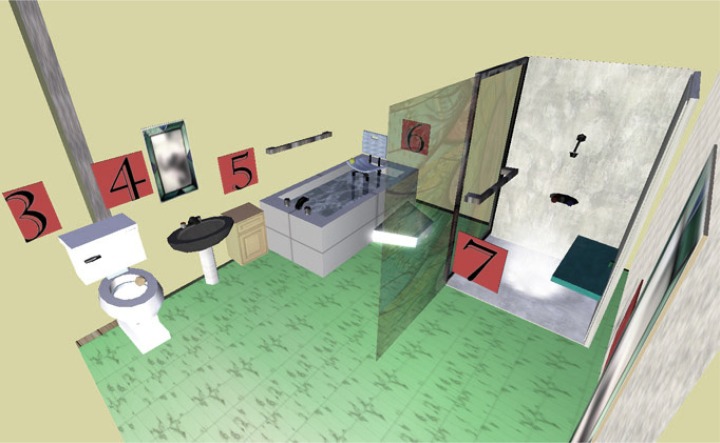
Bathroom in Jefferson’s Adaptation Home.

The Occupational Therapy Center at Jefferson in Second Life^®^ (http://otsecondlife.wordpress.com/ - http://slurl.com/secondlife/Eduisland%20II/203/21/22) has a small home equipped with selected universal design elements and specific types of adapted equipment for persons with disabilities. These include adaptations such as talking medication reminders for persons with low vision, enlarged calendars to help persons with cognitive issues (such as beginning-stage dementia) orient themselves, and a walk-in shower and tub seat for persons with limited mobility. Universal design elements such as lever handles on faucets and a fold-down shower seat demonstrate how easier access and use can be attractive as well as functional (Figure 9). The home includes an on-demand YouTube video about universal design illustrated through the story of a husband and wife who needed to adapt after the woman experienced a stroke. A ‘Healthy Aging’ exhibit alongside the adapted home also provides tips on modifying the environment.

Graduate students from Jefferson are actively involved in the creation of exhibits, as part of their final Masters research projects. Students participate in developing content for exhibits and construct 3D, interactive media to educate the public about health and wellness. This enables them to create a bridge between their “academic” knowledge of home and work adaptation, to the practical and conceptual challenges of conveying this knowledge to others in a way that is both informative and engaging.

The eXtension project at Morrill Virtual State Fair has a large model home with many types of adaptations along with explanations of their purposes. This project is part of a national network, the Cooperative Extension System (Cooperative State Research, Education, and Extension Services, US Department of Agriculture - http://www.csrees.usda.gov/Extension/) designed to provide practical education to communities, particularly in remote and rural areas. Future plans of the eXtension project include homes focused on specific disabilities such as traumatic brain injury that illustrate 25 principles of universal design (eXtension family caregiving SL project -http://extensionfamilycaregivingslp.blogspot.com/).

The focus on home adaptation in Second Life^®^ reflects the increased real life focus on safety and access issues in home environments. Rapid growth in the population of adults over the age of 65 worldwide has created a critical need to address safety and access for older adults [33]. The current movement to foster ‘aging in place’ and the trend for universal design provide support to address these issues. Second Life® model homes thus reflect larger societal trends.

Virtual environments provide an ideal way to educate persons about ways to adapt their home environments for several reasons: they enable 3D, “life-like” models that can be compared and contrasted; individuals can inspect displays at no expense, without consulting an architect or healthcare provider; and the adapted homes are available 24–7 and viewed at the person’s own pace. Moreover, people do not need to physically visit the home, and they can explore and revisit displays very conveniently.

**Figure 10:**
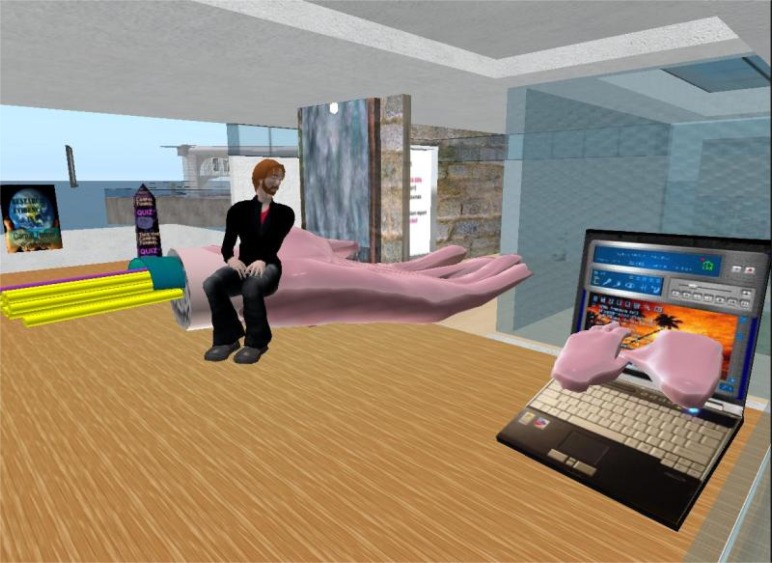
Carpal tunnel exhibit (snapshot by SL:Knoh Oh).

In addition to increasing awareness of home safety and exploring the optimal fit between persons and their living environments, virtual worlds also can be useful for addressing work-related issues. One example is education about ergonomics, or fitting the job to the worker [34], and the risk factors for work-related repetitive strain disorders such as carpal tunnel syndrome. Multiple ways of presenting information can be integrated within virtual environments. For example, at the Occupational Therapy Center at Jefferson in Second Life^®^, exhibits include a 3D model of the hand showing the anatomy of the carpal tunnel (Figure 10), a video with perspectives of a patient with carpal tunnel syndrome and an occupational therapist, slides demonstrating ways to minimize risks, integrated Web links for interactive tutorials and research evidence, and a quiz on proper hand positioning.

While still in its infancy, use of virtual worlds to help visualize ways home environments can be improved and gain greater awareness of ways to minimize risks for work-related injury has strong potential for further development. Immersive environments can complement and further increase awareness of how work and home environments affect safety and health, and may motivate persons to make changes in real life.

## 3. Discussion

3D virtual worlds promise to offer a number of unique pedagogic opportunities—like immersion, visualization, exploratory learning and training, networking and collaboration, co-Web browsing (synchronized, interactive Web browsing by multiple persons), edutainment (e.g., multi-player educational games)/entertainment (e.g., Internet TV/virtual cinema theatres and virtual concerts), *etc.*—that can all be tailored to the n^th^ degree to match the needs of various users, and also linked to real-world and flat Web resources, services and external Internet servers in a variety of novel scenarios that involve data moving both ways between the 3D virtual world platform and the flat Web/rest of the Internet. Our brief tour of some of the early public, environmental and occupational health examples in Second Life® has served to highlight only some of the potentials of these worlds.

### 3.1. What is so Special about 3D Virtual Worlds?

#### 3.1.1. Realism of human and social interactions

Contacts in Second Life^®^ are not just ‘once-only’, though they can definitely be so if a user chooses to limit him/herself to only one-time encounters (e.g., paying anonymous visits to virtual worlds places offering sexual health education for those who prefer to keep their identity private when accessing such material). Many of the communities formed around Second Life^®^ tend to become tightly-knit with time, with people collaborating in groups and getting to know each other better and more personally through their digital identities (and sometimes also by their real identities). Some Second Life^®^ Groups and meetings feel like a real family; bonds are real and so are the losses [35]. The relationships may even extend to real life or make important parts of real life activities, and that is why 3D virtual worlds are sometimes referred to in the literature as 3D real-virtual worlds, since they are and can be a very real part of our real lives [1].

Today’s flat Web allows us to call up “flat” information; a 3D virtual environment allows us to *more naturally experience* and visualise this information in real-time with others and also appreciate their presence around us. Virtual worlds are such an appealing concept to users primarily because of the social ‘co-presence’ of others in these worlds in a very realistic manner.

When people are browsing the flat Web shop of Amazon.com, for example, they cannot see, chat with, and benefit from the experiences/opinions of, other people looking for the same items in real time, as they would do in a supermarket’s aisle in the physical world. But with 3D virtual worlds this is possible [16].

#### 3.1.2. 3D virtual worlds offer a versatile like-real collaboration platform

Unlike conventional video conferencing over the Web, 3D virtual worlds offer strong cognitive cues that enhance collaboration, including 3D spatial audio and avatar lip-synching, bringing emotion/pseudo-body language communication to meetings (thanks to sophisticated, highly customisable avatars and also new technologies like VR-Wear (http://sl.vr-wear.com/about/), as well as a shared pseudo-physical 3D space that can be used in a variety of ways. All of these features bring meetings in 3D virtual worlds one step closer to face-to-face contacts, but in a less “threatening” way that can afford more “protection” for those needing it (Box 1).

Box 1. **Quoted excerpts from [36]**

“Because the full-colour, multifaceted nature of the experience offers **so much more “emotional bandwidth” than traditional Web sites**, e-mail lists and discussion groups, users say **the experience can feel astonishingly real**. Participants develop close relationships and share intimate details even while, paradoxically, remaining anonymous. Some say they open up **in ways they never would in face-to-face encounters** in real support groups, therapy sessions, or even with family and close friends in their true lives.”

An excellent example of an innovative 3D seminar in Second Life^®^ by CIGNA, a health insurance company, has recently been posted on YouTube (http://uk.youtube.com/watch?v=4KRKxv6E8pA), while Sun Microsystem Inc.’s documentation of their own MPK-20 virtual world platform beautifully spells out the unique values of some of the above mentioned affordances of a 3D environment (Box 2).

Humans are spatial beings by nature, inhabiting feature-rich 3-D analogue spaces, so a 3D synthetic space should not be more cognitively demanding from a human-computer interface viewpoint (compared to conventional flat interfaces), if properly designed and presented. In fact, it could even make some presentations that are overly complex in 2D version much less complicated to understand when ported to a more native 3D environment.

Box 2. **Quoted excerpts from http://research.sun.com/projects/mc/mpk20.html by Sun Microsystems, Inc**

**Why 3D for Collaboration? (Sun Microsystems)** “*One question we are frequently asked is why use 3D for a collaboration environment? While it might be possible to build a 2-D tool with functionality similar to MPK-20, the spatial layout of the 3D world coupled with the immersive audio provides **strong cognitive cues that enhance collaboration**. For example, the juxtaposition of avatars in the world coupled with the volume and location of the voices allows people to intuit who they can talk to at any given time. The 3D space provides a natural way to organize multiple, simultaneous conversations. Likewise, the arrangement of the objects within the space provides conversational context. If other avatars are gathering near the entrance to a virtual conference room, it is a good guess that they are about to attend a meeting in that space. It is then natural to talk to those people about the content or timing of the meeting, just as you would if attending a physical meeting. **In terms of data sharing, looking at objects together is a natural activity. With the 3-D spatial cues, each person can get an immediate sense of what the other collaborators can and cannot see***.”

#### 3.1.3. Plasticity and programmability (for user-created content)

Second Life^®^ is also unique because of its very high plasticity, which draws many projects like Play2Train that would otherwise be impossible to develop on other virtual world platforms. This affordance is helping to keep Second Life^®^ strongly desirable, despite the fact that it is largely in the hands of ‘Baby Boomers’ and ‘Generation X’ as far as active users/target audiences go, with younger users statistically remaining much less engaged or interested in the platform [37, 38]. Teens and younger users tend to favour other3D virtual world platforms like vSide (http://www.vside.com/), which are geared more towards entertainment and youth culture, but these worlds offer far much less plasticity and programmability compared to Second Life^®^.

Finally, it is the unique *combination* of all of the above features and affordances under one platform that makes 3D virtual worlds very special (realism of human and social interactions; co-presence; versatile, like-real (and in real-time) collaboration and presentation/visualization platform with 3D space and many important cognitive cues; and plasticity).

### 3.2. The Web 3D of the Future

Instead of a conventional discussion of the current limitations of 3D virtual worlds, an exercise we have already covered in previous publications, e.g., [14–17], we will try in the following section to present those same limitations in a more positive way, as ‘emerging and expected future development directions’ of 3D virtual worlds.

These are early days of 3D virtual worlds, similar in some way to the beginnings of the World Wide Web (WWW), when much of the content during those days comprised mainly of a straightforward port to the Web of digitized paper brochures and printed material in the form of long pages of text (formatted like a book), interspersed with some low-quality graphics (because of image file size/download bandwidth constraints at that time), and some links between the pages.

It then took almost a decade to realize the ideas we are seeing today in Web 2.0 applications [39], yet Web 2.0 is not at all new or the product of the past few years. It is a Web evolution rather than a platform revolution. The concepts underpinning its development (e.g., ‘people’s Web’, ‘read-write Web’, ‘user-generated content and mashups’) date back to the early 1990s when Sir Tim Berners-Lee conceived the WWW [40] and even before those days. (Most of Web 2.0’s boasted multi-user affordances and concepts of social networking, voting, content sharing/collaboration, *etc.* were also present in some primitive form in the pre-WWW, 4,800/9,600 bps modem BBSes (Bulletin Board Systems) and online services like CompuServe.) This “gestation” decade of the so-called Web 2.0 was essential for: (1) the Web to build a massive user base and become more popular, and (2) for the technology enabling this to evolve, which were the prerequisites of the social Web we have today.

Time was also required to convince sceptics about the utility of the Web, which also faced opposition during its early years by luddites and ‘doubting Thomases’, who were questioning its utility, until they were able to see with their own eyes its later successes. Hansen [4] compared this kind of opposition to introduction of the stethoscope in the 19^th^ century: “*With any innovative technology there are critics who debate its usefulness. However, one recalls when critics questioned the validity and reliability of the stethoscope invented by Laennec in 1816 and how today it is second nature to use this assessment tool.*”

Similarly, 3D virtual worlds are not a new concept (though the current generation of 3D virtual worlds is relatively young, about five years old). The US Department of Defense has been using virtual worlds since the 1990s [15] (see also http://research.microsoft.com/scg/vworlds/vworlds.htm and http://www.dipity.com/user/xantherus/timeline/Virtual_Worlds/), and there are still today those who perceive little value in these worlds and cannot see beyond the current technology limitations of these platforms. There are, however, others who recognise 3D virtual worlds as a true Web revolution rather than a mere technology evolution, perhaps because of their very different interfaces (immersive 3D vs. flat 2D) [41, 42].

To the latter group, non-gaming 3D virtual worlds are part of the future 3D Internet or Web 3D [15, 19], though of course not in their current (2008) form or technology, (which is also still largely proprietary, with the main exception of OpenSim (http://www.opensimulator.org/), and thus not fully compatible with the open standards spirit of the Web that was crucial for its development).

Professor Miklos Sarvary, Director of the Centre for Learning Innovation at Insead, has drawn parallels between the life cycle of broadcasting and the Internet. Just as radio gave way to the more immersive experience of TV, today’s flat Web sites will morph into more interactive, immersive multiuser experiences in which users can see and interact with each other in more natural ways [43].

It is predicted that, within 7–10 years, the dominant Internet interface is likely to be the 3D ‘Metaverse’, the next-generation, high-definition media-rich Web 3D that will gradually absorb, and seamlessly integrate with (not fully replace), today’s essentially flat World Wide Web and its early 3D applications like Google Earth and Second Life^®^[15, 19, 41].

The European Commission is even officially recognising the 3D Internet as a strategic research direction, as seen, for example, in their Seventh Framework Programme (FP7) call for proposals issued on 19 November 2008 [44].

#### 3.2.1. Standards for Web 3D

Standards are cornerstones of the Web, including Web 3D. Without such standards in place, all current 3D virtual world developments and investments are not sustainable in the long run. Today’s virtual worlds industry is very fragmented, operating more like the computer gaming industry than the Internet industry. Each developer, be it private or open source, is developing its own server and client architectures, and rules of engagement. However, the common public interest calls for a connected system like the flat Web is on today’s Internet, where different forces can innovate in particular spots of the value chain [45]. In this respect two developments are worth noting in the context of 3D virtual worlds. The first one is the emerging International Organization for Standardization (ISO) MPEG-V standard (‘V’ for ‘Virtual worlds’), which is looking into establishing formal specification sets for bridging the gaps (i.e., interoperability) within different virtual worlds and between virtual and real/physical worlds like sensors worn on the body or in the environment [46] (final standard ratification is expected by October 2010 – see also http://yesha.sivan.googlepages.com/VirtualWorldsSOSv01-03a.pdf). Interestingly, the MPEG-V requirements specification document approved by ISO in 2008 [46] features two health-related ‘use-cases’, one about AAL (Ambient Assisted Living) for older people and the other focused on disaster management.

The second standards-related development that is highly relevant to Web 3D is COLLADA (http://www.collada.org/), a COLLAborative Design Activity for establishing an interchange file format for interactive 3D applications, managed by a not-for-profit technology consortium, the Khronos Group. COLLADA is currently supported in Google Earth (see, for example, Ancient Rome 3D in Google Earth at http://www.google.com/intl/en/press/annc/20081112_earthrome.html) and Google 3D Warehouse (http://sketchup.google.com/3dwarehouse/), but sadly not in Second Life^®^.

#### 3.2.2. Technology trends and maturation

The ubiquitous 3D Internet will fully support mobile devices (we have already started to see this in May 2008—see http://www.vollee.com/secondlife). The technology powering virtual worlds and Web 3D, e.g., GPUs (Graphics Processing Units) and Internet infrastructure/bandwidth, will continue to get better performance wise at same price of, or even cheaper than previous generations of the same product.

Better virtual world platform scalability and reliability are also expected, as well as better user interface accessibility and usability. 3D virtual worlds are rapidly getting more accessible and user friendly (even for people with cognitive and/or physical disabilities). Many new input and interaction modalities are being used and developed, e.g., speech-to-text /text-to-speech and on-the-fly multilingual translation, novel uses of the popular Wiimote (http://wii.com/), CamSpace (http://www.camspace.com/), Hands Free 3D (http://www.handsfree3d.com/), 3D SpaceNavigator (http://www.3dconnexion.com/solutions/secondlife.php), 3D Force Mouse/3D Touch (http://www.anarkik3d.co.uk/ -http://home.novint.com/), multi-touch screens, and even technologies for the blind and deaf to navigate these worlds (http://news.bbc.co.uk/2/hi/technology/6993739.stm - http://news.bbc.co.uk/1/hi/technology/6993326.stm).

We will also witness increasingly better immersiveness (realism) of Web 3D experiences over the coming years [42], as Internet bandwidth and infrastructure expand, and as GPUs continue to get more powerful [47, 48], with more stream processors to crunch the most complex graphics and demanding scenes. Support for true 3D Stereo Vision is already found in today’s hardware (http://www.nvidia.com/object/3d_stereo.html), and some of the latest software graphics engines around like Ubisoft’s Scimitar engine (http://en.wikipedia.org/wiki/Scimitar_%28game_engine%29) are setting new levels or ‘gold standard’ for what can be achieved in terms of realism and immersiveness in a 3D virtual environment. The Scimitar engine, to use the same example, supports dynamic world loading/full roaming, sandbox environments; advanced realistic physics; full avatar animation (complex and fine movements); complex weather lighting and realistic dynamic shadows of both static and moving objects; all while being extremely efficient in terms of computing resources it needs to run on highest quality settings. (For readers wanting to try it, the Scimitar engine powers Assassin’s Creed, a 2007/2008 game developed by Ubisoft Montreal, as well as some other recent games by Ubisoft.) Another equally-advanced graphics engine worth noting is Nurien 3D social networking platform (http://www.nurien.com/ - video: http://www.youtube.com/watch?v=MoQh4R1-Vjs). Second Life^®^ is very much lagging behind by comparison.

#### 3.2.3. Skills for Web 3D

With all these technological developments, designers and developers of Web 3D presences must make sure the message of their 3D experiences does not become eclipsed by any cutting-edge technology used to deliver it. Technology needs to be transparent, almost invisible. You feel its presence, but you do not see it. It has to prompt curiosity but remain discrete, so that one can fully focus on the message of the 3D experience. This is key to successful immersiveness (‘suspension of disbelief’).

It is also evident that Web 3D requires a different, yet complementary, set of skills to those needed to develop 2D (flat) Web portals. What the 3-D Web demands is cinematographers, story-tellers, actors and directors who can get people involved in plots and incentive-based structures that are familiar from everyday life, movies and video games, as one report by McKinsey & Co mentions [49] (see also http://uk.youtube.com/watch?v=Qtn5wcDA0is for a ‘behind the scenes’ overview of the development of a complex health-related presence in Second Life^®^).

### 3.3. Final Notes—Instantly Impacting Real Lives

There are still many untapped potentials and affordances of 3D virtual worlds that are yet to be explored, as the technology matures further over the coming months and years. It is very reassuring to see reputable US federal agencies like the CDC and NASA build significant presences in 3D virtual worlds today, but those excellent examples we have seen so far are just the beginning of an even more exciting journey towards the full realization of Web 3D. For example, an unpublished research protocol by Kamel Boulos proposed in 2008 a novel 3D experience for behaviour and lifestyle modification, combining concepts from Ohio University’s ‘*The Nutrition Game: A Day of Food Choices*’ in Second Life^®^ (http://www.youtube.com/watch?v=nLhNNYRJwJ4 and [14]) with a person’s actual grocery and food shopping in a Second Life^®^ v-supermarket interfacing with an existing flat Web supermarket (a v-shopping/v-commerce mashup—‘v’ for ‘virtual’, but the transactions are very real and goods get dispatched to the shopper’s physical home address). Since education about diet and nutrition is of prime importance to, for example, diabetics, having such a “clever” guide like in the Ohio nutrition game directly linked to/embedded in the actual shopping for food and groceries can help transform health education into positive shopping/eating behaviour and lifestyle changes. The whole shopping experience, education and advice provided can be further individualized and tailored based on the person’s health profile and clinical data/trends, as well as any recorded clinician’s recommendations in relation to these details. This ‘shopping healthily’ example can be further enhanced with a Second Life^®^-assisted controlled physical exercise programme, in which the person receives stimulating visual feedback from the 3D immersive world while exercising or jogging on a treadmill (this is achieved by connecting special controllers like the Nintendo Wii controller to the machine running Second Life^®^ - see http://www.youtube.com/watch?v=c1wtAlAYiUE). The end result will be better health and a tangible positive impact on real lives.
